# Impact of Screening on Mortality for Patients Diagnosed with Hepatocellular Carcinoma in a Safety-Net Healthcare System: An Opportunity for Addressing Disparities †

**DOI:** 10.3390/cancers16223829

**Published:** 2024-11-14

**Authors:** Kalyani Narra, Madison Hull, Kari J. Teigen, Vedaamrutha Reddy, Jolonda C. Bullock, Riyaz Basha, Nadia Alawi-Kakomanolis, David E. Gerber, Timothy J. Brown

**Affiliations:** 1 John Peter Smith Health Network, Fort Worth, TX 76104, USA; 2 Department of Internal Medicine, Burnett School of Medicine at Texas Christian University, Fort Worth, TX 76104, USA; 3 Texas College of Osteopathic Medicine, Fort Worth, TX 76107, USA; 4 University of Connecticut, Storrs, CT 06269, USA; 5 Division of Hematology-Oncology, Department of Internal Medicine, University of Texas Southwestern Medical Center, Dallas, TX 75390, USA; 6 Harold C. Simmons Cancer Center, University of Texas Southwestern Medical Center, Dallas, TX 75390, USA; 7 Peter O’Donnell Jr. School of Public Health, University of Texas Southwestern Medical Center, Dallas, TX 75390, USA

**Keywords:** hepatocellular carcinoma, screening, outcomes

## Abstract

**Purpose:** We describe the impact of screening on outcomes of patients diagnosed with hepatocellular carcinoma (HCC) in an urban safety-net healthcare system compared to a non-screened cohort diagnosed with HCC. **Methods:** Patients diagnosed with HCC at John Peter Smith Health Network were identified by querying the hospital tumor registry and allocated to the screened cohort if they had undergone any liver imaging within one year prior to HCC diagnosis, while the remainder were allocated to the non-screened cohort. Kaplan–Meier methods and log-rank tests were used to compare 3-year survival curves from an index date of HCC diagnosis. Cox proportional hazard models were used to calculate unadjusted and adjusted hazard ratios (HRs) and 95% confidence intervals (CIs). The Duffy adjustment was used to address lead-time bias. **Results:** A total of 158 patients were included (n = 53 screened, n = 105 non-screened). The median overall survival (OS) for the screened cohort was 19.0 months (95% CI: 9.9–NA) and that for the non-screened cohort was 5.4 months (95% CI: 3.7–8.5) [HR death (non-screened vs. screened) = 2.4, 95% CI: 1.6-3.6; log rank *p* < 0.0001]. The benefit of screening remained after adjusting for lead-time bias (HR 2.19, 95% CI 1.4–3.3, *p* = 0.0002). **Conclusions:** In an urban safety-net population, screening for HCC was associated with improved outcomes compared to patients diagnosed with HCC outside of a screening protocol.

## Simple Summary

Hepatocellular carcinoma (HCC) is a common and deadly cancer, almost always arising from a background of chronic liver disease. Patients at high risk for developing HCC are recommended to undergo semiannual screening for HCC with serum alpha-fetoprotein and liver ultrasound. However, for patients of lower socioeconomic status, adherence to this recommended screening program can be challenging. Here, we describe outcomes of patients diagnosed with HCC seen primarily in a county safety-net healthcare system. Patients were analyzed by whether or not they were screened for HCC or diagnosed incidentally. We find that patients diagnosed as part of a screening regimen have improved overall survival and that this improvement persists after adjustment for lead-time bias. Dedicating resources to HCC screening can meaningfully address existing disparities for patients at risk for development of HCC.

## 1. Introduction

Hepatocellular carcinoma (HCC) is a deadly and increasingly common cancer of the liver [1]. In addition to the typically aggressive behavior of HCC, it almost always develops from a background of chronic liver disease, which may limit treatment options for patients with HCC [2]. Many of these chronic conditions, including alcohol use disorder, viral hepatitis (B or C), and metabolic-dysfunction-associated steatotic liver disease (MASLD), are known to disproportionately affect individuals of lower socioeconomic status (SES). Because these populations are already at high risk of disparate outcomes, this perpetuates racial, ethnic, and economic disparities that are observed throughout clinical oncology [2,3,4,5]. 

Individuals of lower SES are less likely to participate in recommended cancer screening exams compared to the general population and are more likely to present with severe disease for numerous reasons [5]. These include less access to care and higher prevalences of alcohol use disorder, non-alcoholic fatty liver disease, hepatitis C virus, hepatitis B virus, and autoimmune hepatitis [6,7,8,9]. For patients with cirrhosis at high risk of developing HCC, screening with an ultrasound of the liver and serum alpha fetoprotein (AFP) is recommended every 6 months [10,11,12,13,14,15,16,17]. Such a strategy produces a sensitivity of 61% and specificity of 92% for the early diagnosis of HCC and is currently the most cost-effective strategy available [10,12,18]. The goal of such screening is to identify patients with small, clinically quiescent tumors amenable to curative-intent modalities—transplantation, resection, ablation, or embolization. However, significant disparities in access to HCC screening exist, such as lower individual resources, busier emergency rooms that contribute to delays in initial treatment, and less access to specialized services such as liver transplants that all contribute to later presentation to medical attention [19,20,21,22]. Further, several factors can subsequently impact the effectiveness of HCC screening, including male gender, higher body mass index, more advanced cirrhosis, non-alcoholic steatohepatitis cirrhosis, and inpatient status [23,24].

Because of these recognized disparities in completing screening and the potential downstream effects on outcomes of patients with HCC, we sought to describe the impact of the diagnosis of HCC within a screening program compared to outside of a screening program on mortality in patients diagnosed with HCC in a large urban safety-net healthcare system that provides care to individuals of lower SES.

## 2. Methods

### 2.1. Study Setting and Institutional Review Board Oversight

This study was conducted at the John Peter Smith Health Network (JPS) in Fort Worth, Texas—the safety-net hospital and comprehensive healthcare system serving 179,223 patients per year among a potential catchment of 2.1 million people in Tarrant County. This hospital serves a high proportion of the country’s medically underserved population and is an ideal setting for describing the impact of disparities on outcomes for patients with HCC. This study was approved by the North Texas Regional Institutional Review Board and granted a waiver of consent (IRB #1918962-1). Portions of this study were presented in abstract form at the American Association for Cancer Research (AACR) Annual Meeting, 14–19 April 2023, Orlando, FL, USA, and the 2024 ASCO Quality Care Symposium 27–28 September 2024, San Francisco, CA, USA [25,26].

### 2.2. Study Procedures, Clinical Data Collection, and Characterization

This was a retrospective cohort study of all patients diagnosed with HCC at JPS between 1 January 2018 and 31 March 2021, with the intent of having a three-year follow-up for all patients. Patients with a new diagnosis of HCC were identified from the JPS Tumor Registry, an established registry that captures all patients with newly diagnosed cancer within the health system as required by law. Patients were included if they were aged 18 years or older with a confirmed diagnosis of HCC during the study period. HCC could be diagnosed by radiographic determination in the appropriate clinical context or by tissue diagnosis [10,27]. Patients who did not receive longitudinal cancer care at JPS were excluded. Charts of patients meeting the criteria for inclusion were reviewed for the following: age at diagnosis, race/ethnicity, sex, insurance status, alcohol use disorder, Hepatitis C (HCV) and Hepatitis B (HBV) status, cirrhosis, and AFP level. Insurance status at diagnosis was characterized as private, public, and uninsured. AFP levels were categorized as follows: 0–399 ng/mL and ≥400 ng/mL [28]. Alcohol use disorder was captured using the social history section of the electronic medical record (EMR). Hepatitis C (HCV) and Hepatitis B (HBV) status were determined based on positive serologic testing. Cirrhosis was identified through imaging findings consistent with cirrhosis or histopathologic confirmation. For the purposes of cohort allocation, we recorded whether patients had undergone any radiographic screening for HCC (ultrasound or computed tomography [CT]) within the one year before HCC diagnosis. Although the recommended screening interval is every 6 months, patients were allocated to the screened cohort if they underwent radiographic screening within one year prior to HCC diagnosis if the reason for the test order stated “screening” or “surveillance”, recognizing that the unique challenges of navigating the safety-net health system may contribute to delays and adherence to strict screening protocols. Those diagnosed with HCC without a screening exam record within 1 year were allocated to the non-screened cohort. The primary endpoint of interest was mortality from diagnosis. Patients were followed from the index date of diagnosis until death or censored at the date of the last known encounter prior to 1 April 2024.

### 2.3. Statistical Analysis

Summary statistics consist of frequencies and percentages for categorical data and means and standard deviation for continuous data. Statistical differences in demographic and clinical characteristics by screening status were determined by the *t*-test for continuous variables and by the chi-square or Fisher’s exact test for categorical variables. A survival time cut-off point of 3 years was used for analysis to ensure all patients had the same window of observation. Kaplan–Meier analysis was used to illustrate and compare 3-year survival curves by screening status. A log rank test was used to assess differences in survival curves. The Cox proportional hazard model was performed to examine the association of screening status and three-year all-cause mortality, adjusting for covariates. To establish a full multivariate model, all covariates underwent univariate pre-filtering. All available items were analyzed via a univariate Cox proportional hazard model and items with a *p*-value of ≤0.2 were analyzed in the multivariable model. Backwards stepwise covariate selection was used on the items identified via pre-univariate filtering to construct the most parsimonious model by selecting the model with the lowest Akaike information criterion (AIC), a measurement of model goodness of fit. The significance level was set at 2-sided *p* < 0.05 in all statistical tests.

To adjust for lead-time bias associated with screening, we performed sensitivity analyses using the Duffy adjustment [29,30]. Briefly, the survival time in the screening cohort was adjusted by an exponential distribution of the sojourn time—the time between when a tumor can be detected on screening exams and when it can become clinically apparent [29,30]. In HCC, prior studies have proposed a sojourn time of 70–140 days. Thus, we performed two separate sensitivity analyses with the Duffy adjustment using a sojourn time of 70 days and 140 days [14,15,31,32,33]. Because the goal of this was a sensitivity analysis, no full Cox proportional hazard model was constructed after Duffy adjustment. All analyses were performed using SAS v.9.4 (Cary, NC, USA).

## 3. Results

A total of 158 patients were included in the analysis. The mean age was 62 years (standard deviation: 8.8), 78% were male, 34% were non-Hispanic white, and 8% had private insurance. Of these patients, 53 (34%) were detected through radiographic screening. Of the 53 patients in the screening cohort, 48 had undergone screening exams within 6 months of diagnosis of HCC. Baseline characteristics for screened and non-screened cohorts are summarized in Table 1.

**Figure 1:**
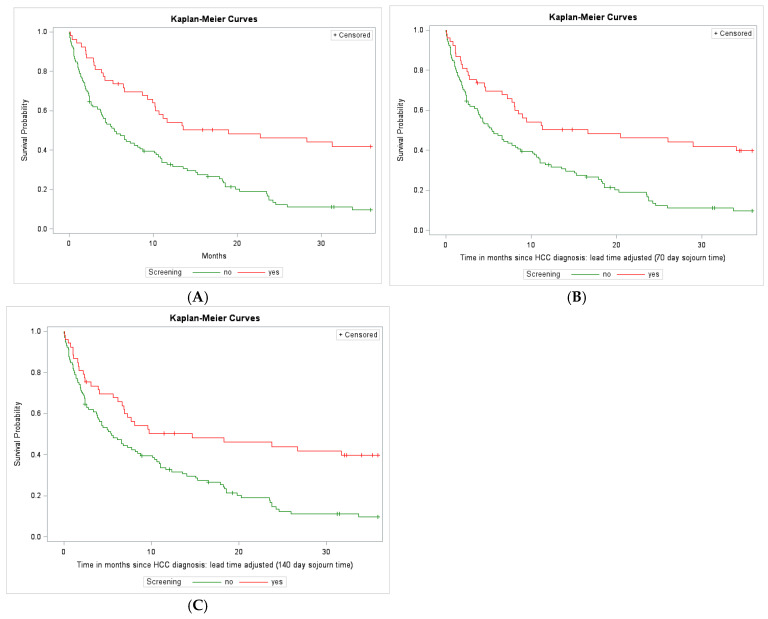
(**A**) Kaplan–Meier curve by screening status of 158 patients. The median overall survival for patients in the screened cohort was 19.0 months (95% CI: 9.9—NA), while the median overall survival for patients in the non-screened cohort was 5.4 months (95% CI: 3.7, 8.5) (log-rank *p* ≤ 0.0001). (**B**) Kaplan–Meier curve by screening status of 158 patients, adjusted for a presumed lead time (mean sojourn time of 70 days). The median survival for patients in the screened cohort was 17.0 months (95% CI: 7.8—NA). (**C**) Kaplan–Meier curve by screening status of 158 patients, adjusted for a presumed lead time (mean sojourn time of 140 days). The median survival for patients in the screened cohort was 15.0 months (95% CI: 6.6—N.A).

The median overall survival was 8.7 months (95% confidence interval (CI): 5.4–11.0). Within the first 3 years of follow-up, 27 patients (17%) survived, 121 patients (77%) died, and 10 (6%) patients were lost to follow-up. The median overall survival for patients in the screened cohort was 19.0 months (95% CI: 9.9—NA), while the median overall survival for patients in the non-screened cohort was 5.4 months (95% CI: 3.7–8.5) (log-rank *p* ≤ 0.0001) (Figure 1A).

The Cox proportional hazard model showed a statistically significant difference in hazard ratio of death in the first three years for those in the non-screened cohort compared to patients in the screened cohort (HR: 2.4; 95% CI: 1.6, 3.6; *p* ≤ 0.0001) (Table 2). After adjusting for ALBI grade, AFP group, hepatitis C, insurance status, and treatment received, there was a statistical difference in hazard of death at three years (aHR: 1.9; 95% CI: 1.2, 3.1; *p* = 0.009) (Table 2), favoring the screened cohort.

### Lead-Time-Bias-Adjusted Overall Survival

We calculated the lead time with an assumption of two different HCC sojourn times, 70 and 140 days. Figure 1B,C show the adjusted Kaplan–Meier curves. Both analyses showed a statistically significant longer survival in the screened cohort. The adjusted median survival for the screened cohort with a mean sojourn of 70 days was 17.0 months (95% CI: 7.8—NA). The adjusted median survival for the screened cohort with a mean sojourn of 140 days was 15.0 months (95% CI: 6.6—NA). Both were compared to the non-screened cohort. This was statistically significant in the univariate hazard model, as shown in Table 3.

## 4. Discussion

For those of limited socioeconomic means, publicly funded safety-net healthcare systems can provide affordable, accessible, and cutting-edge medical care that spans the screening, diagnosis, and treatment spectrum and can mitigate disparities in oncology [34]. These services have particular importance for patients with HCC, where multidisciplinary care has long been recognized to significantly improve outcomes, including in safety-net settings [10,35,36,37]. Despite challenges to delivering guideline-concordant screening for patients in a safety-net setting, dedicated programs and systematic improvements can positively impact outcomes for these patients [34,38,39,40]. A successful description of outcomes will allow for program development specifically targeting intervention methods, expanding clinical management, and ultimately improving the prognosis for patients affected by HCC [41,42,43].

In this three-year follow-up analysis of a population of patients diagnosed with HCC in a large urban safety-net healthcare system, we observed that patients who underwent HCC screening within one year prior to the diagnosis of HCC experienced improved mortality compared to those patients diagnosed with HCC outside of a screening program. These findings remained significant after adjusting for key baseline covariates (AFP group, hepatitis C, insurance status, race, and ethnicity) and after adjusting for the potential lead-time bias associated with screening.

The benefit of screening for HCC in patients at high risk for HCC is widely recognized [32,33,44]. Further, screening for HCC in patients at high risk for developing HCC is more cost-effective in terms of quality-adjusted life years than not screening for HCC in patients with compensated cirrhosis, owing to an association with earlier diagnosis and improved survival [18]. However, with these findings in our report, we propose that additional resources should be directed to these underserved individuals to ensure the opportunity to participate in recommended screening for HCC. Currently accepted screening protocols for patients at risk for developing HCC include tandem AFP and ultrasound exams every 6 months, a protocol that was derived from a prospective trial demonstrating a survival benefit in patients with hepatitis B infection and further supported by several cohort studies and meta-analyses [10,13,15,16,17,43]. However, clearly demonstrating the impact of screening in a retrospective database remains a challenge due to the inherent lead-time bias associated with screening. Statistical methods for adjusting for lead time may provide some insight into the impact of screening while allowing for some adjustment of lead time [29,30]. One such acceptable method for adjusting for lead-time bias in the screening arm is the Duffy adjustment [29,40]. In our database, the benefit of screening was still apparent following several sensitivity analyses using the Duffy adjustment to adjust for sojourn times of 70 and 140 days in the screened cohort [14,15,31,32,33]. Proportionally more patients in the screening cohort received locoregional treatments (resection, transarterial chemoembolization, or transarterial radioembolization) and more patients in the non-screened cohort received either no treatment or systemic therapy as the initial treatment, although these differences did not reach statistical significance. These observations suggest that screening may lead to earlier diagnosis and an opportunity for earlier interventions that can alter long-term outcomes for these patients.

In our study, we observed a high proportion of Hispanic and Black patients diagnosed with HCC, although the distribution was not different between groups. Most patients in our study had a diagnosis of cirrhosis and a high proportion endorsed a history of alcohol use or had evidence of active or prior hepatitis C infection, consistent with prior studies in this patient population [38]. We note that the proportion of patients with cirrhosis, alcohol use, and hepatitis C infection was similar across groups. However, in the present study, the overall survival for the patients in both arms of this study was somewhat shorter compared to other studies of HCC screening in the United States [16]. Yet, the magnitude of the benefit of screening as measured by the hazard ratio was consistent with prior reports [16,17]. These findings are likely somewhat reflective of the high proportion of patients with evidence of advanced liver disease as measured by the ALBI grade. In particular, patients in the non-screened cohort had evidence of more severe liver dysfunction at the time of HCC diagnosis compared to the screened cohort. Similarly, more patients in the non-screened cohort had a baseline AFP ≥400 ng/mL, potentially reflecting an increased tumor burden when HCC is diagnosed outside of a screening protocol compared to diagnoses made within the context of screening protocols.

We also observed an association towards better survival outcomes in a univariate Cox regression in Hispanic patients included in our study. In the United States, the incidence of HCC has been increasing in the Hispanic population, which has been attributed to the increasing prevalence of metabolic-associated steatotic liver disease [9,45,46,47,48]. The impact of Hispanic ethnicity on survival has infrequently been described in prior studies examining the relationship between race, ethnicity, and outcomes in patients with HCC [48]. To our knowledge, this positive association between Hispanic ethnicity and improved survival in HCC has not previously been reported. However, this phenomenon, called the “Hispanic paradox”, is a recognized epidemiologic phenomenon within the United States and has been reported in other tumor types and medical conditions [49,50,51,52]. The observation that patients of Hispanic ethnicity have improved outcomes compared to patients of other ethnicities has been attributed to a supportive home environment, health behaviors, and genetic factors [49,53,54]. Additional study will be needed to understand reasons for the improved survival among the Hispanic population in our cohort to identify modifiable factors or behaviors that can be applied to or adapted by other groups.

Importantly, this study must be interpreted in the context of its limitations. First, this was a single-institution study. Although JPS serves a large and diverse patient population, some outcomes observed may reflect local practice patterns and specific patients treated at JPS. Second, a complete HCC staging with Barcelona Clinic Liver Cancer and/or Tumor, Node, and Metastasis staging was not available and thus we cannot assess potential imbalances in the stage of HCC between arms. We lack data on macrovascular invasion and extrahepatic spread, important prognostic factors when considering the approach to the treatment of HCC. Similarly, complete descriptions of the severity of liver dysfunction with the Model for End-Stage Liver Disease (MELD) and Child–Pugh scores were not feasible with the available data. This difficulty is a recognized limitation in retrospective research in cirrhosis and HCC, which frequently lacks the laboratory values and/or subjective assessments included in these scores [55]. Further, we did not collect data regarding the treatment of chronic liver disease or other potential comorbidities for patients included in our study. Treatment of the underlying liver disease is recognized to improve outcomes for patients subsequently diagnosed with HCC; however, the response to treatment in the advanced setting does not differ between etiologies [56,57]. However, we were able to completely describe the liver disease severity in our cohort using the ALBI score, which is recognized to correlate with prognosis in HCC [58,59,60]. We did not perform next-generation sequencing on tumor samples from patients within our analysis diagnosed with HCC and thus cannot rule out the possibility of an imbalance between cohorts. Next, most of these study data originated in the era before immune checkpoint inhibitors were routinely used for the first-line management of advanced HCC [61,62]. Nearly all of the patients in our study treated with systemic therapy received either sorafenib or lenvatinib, accepted first-line treatment options at the time [63,64]. It is possible with the emergence of combination atezolizumab and bevacizumab, combination durvalumab and tremelimumab, and pembrolizumab that the magnitude of benefit may change in the future [61,62,65]. Importantly, rates of receipt of systemic therapy as initial treatment for HCC were not different between the cohorts. We acknowledge that we could not individually calculate tumor doubling time for patients included in this study, instead adopting the sojourn time from prior publications in HCC screening [14,15,31,32,33]. A recent publication used individual tumor doubling time to adjust for lead time and length-time bias among patients who had two imaging studies >30 days apart without treatment for HCC, finding similar results for the impact of screening as our study although with a much longer overall survival in the cohort [40]. While we did not specifically measure differences with regard to access to healthcare utilization and thus cannot rule out the possibility of systematic barriers to care that affected the non-screened cohort disproportionately, the safety-net nature of JPS likely lowers the barrier to access to care as much as is feasible. Thus, the possibility of disproportionate barriers to healthcare utilization is possibly minimized. Lastly, JPS, like many safety-net healthcare systems, does not have the facilities to perform liver transplantation, a potentially curative procedure that can be offered as a curative modality to patients with small tumors or with advanced liver disease prior to development of HCC. However, patients with private or public insurance may have an option to be referred for liver transplant [10]. The availability of liver transplantation could significantly impact long-term outcomes for these patients, particularly those with small HCC tumors identified by screening programs [10]. Thus, the results reported here should be interpreted in the context of this important caveat, although it should be recognized that transplant receipt for HCC necessitates a 6-month period of tumor stability prior to transplant receipt [10,66].

## 5. Conclusions

We find that engagement in a screening program for HCC in a public safety-net healthcare system was associated with improved outcomes compared with patients diagnosed with HCC outside of a screening program. This benefit remained significant following adjustment for key baseline covariates and in sensitivity analyses adjusting for potential lead-time bias. Altogether, this implies that the earlier diagnosis and institution of treatment can positively improve survival for patients with newly diagnosed HCC in a population of patients evaluated at a safety-net hospital. The development of screening programs in accordance with accepted guidelines with outreach to high-risk individuals represents an area of high-value care that is worth investment by safety-net hospitals.

## Figures and Tables

**Table 1:** Characteristics of 158 patients diagnosed with hepatocellular cancer according to screening status. Note: percentages do not include missing data unless otherwise stated. ^1^ *t*-test, ^2^ chi-square, ^3^ Statistical test excluded “missing”, ^4^ Fisher’s exact test.

Characteristic	Screened (n = 53)	Not Screened (n = 105 )	*p*
	Mean ± SD or n (%)		
**Age at Diagnosis ^1^**	61 ± 8.6	62 ± 9.0	0.54
**Alcohol ^2,3^**			0.43
Yes	35 (66)	62 (59)	
No	18 (34)	42 (40)	
Missing		1 (1)	
**ALBI grade ^4^**			0.001
A1	7 (13)	3 (3)	
A2	28 (53)	37 (35)	
A3	18 (34)	65 (62)	
**AFP group ^2,3^**			0.01
<400 ng/L	35 (66)	48 (46)	
≥400 ng/L	16 (30)	54 (51)	
Missing	2 (4)	3 (3)	
**Gender ^2^**			0.36
Male	39 (74)	84 (80)	
Female	14 (26)	21 (20)	
**Cirrhosis ^4^**			0.04
Yes	52 (98)	92 (88)	
No	1 (2)	13 (13)	
**Hepatitis B ^4^**			0.76
Yes	5 (9)	8 (8)	
No	48 (91)	96 (91)	
Missing		1 (1)	
**Hepatitis C ^2,3^**			0.54
Yes	41 (77)	75 (71)	
No	12 (23)	28 (27)	
Missing	0	2 (2)	
**Insurance Type ^4^**			0.18
Private	5 (9)	8 (8)	
Public	28 (53)	38 (36)	
Uninsured	17 (32)	51 (49)	
Missing	3 (6)	8 (8)	
**Race and Ethnicity ^2^**			0.23
Non-Hispanic White	21 (40)	33 (31)	
Non-Hispanic Black	12 (23)	36 (34)	
Hispanic	15 (28)	32 (30)	
Other	5 (9)	4 (4)	
**Treatment Received ^4^**			0.08
None	19 (36)	51 (49)	
Resection	3 (6)	2 (2)	
Locoregional	27 (51)	34 (32)	
Systemic	3 (6)	12 (11)	
Missing	1 (2)	6 (6)	

**Table 2:** Association between clinical and demographic characteristics and 3-year all-cause mortality following hepatocellular carcinoma diagnosis: Cox proportional hazard regression models and Univariable Cox Regression Analysis of demo-clinical characteristics for survival of hepatocellular cancer diagnosis. ^1^ Adjusted for ALBI grade, AFP group (0–399 ng/mL vs. ≥400 ng/L), hepatitis C insurance status (private, public, uninsured), and initial treatment (none, resection, locoregional, systemic).

Characteristic	Univariate			Multivariable ^1^		
	HR	95% CI	*p*	HR	95% CI	*p*
**Age at Diagnosis**	1.01	(0.99, 1.03)	0.43			
**Alcohol**						
No	0.99	(0.69, 1.43)	0.96			
Yes	Reference	Reference				
**ALBI grade**						
A1	0.15	(0.05, 0.48)	0.0013	0.000	0.000	0.98
A2	0.39	(0.27, 0.57)	<0.0001	0.42	(0.27, 0.64)	<0.0001
A3	Reference	Reference		Reference	Reference	
**AFP Group**						
0–399 ng/L	0.36	(0.25, 0.51)	<0.0001	0.40	(0.26, 0.61)	<0.0001
≥400 ng/L	Reference	Reference		Reference	Reference	
**Cirrhosis**						
No	1.2	(0.66, 2.2)	0.56			
Yes	Reference	Reference				
**Gender**						
Female	0.84	(0.54, 1.3)	0.44			
Male	Reference	Reference				
**Hepatitis B**						
No	1.5	(0.75, 3.1)	0.25			
Yes	Reference	Reference				
**Hepatitis C**						
No	0.76	(0.5, 1.2)	0.20	1.06	(0.66, 1.7)	0.80
Yes	Reference	Reference		Reference	Reference	
**Insurance Type**						
Private	Reference	Reference		Reference	Reference	
Public	2.0	(0.92, 4.5)	0.08	1.89	(0.73, 4.9)	0.1902
Uninsured	2.0	(0.91, 4.4)	0.08	1.53	(0.59, 3.9)	0.3823
**Race and Ethnicity**						
Non-Hispanic White	Reference	Reference				
Non-Hispanic Black	0.78	(0.5,1.2)	0.27			
Hispanic	0.78	(0.5, 1.2)	0.28			
Other	0.62	(0.26, 1.5)	0.27			
**Treatment Received**						
None	Reference	Reference		Reference	Reference	
Resection or Locoregional	0.26	(0.17,0.39)	<0.0001	0.34	(0.22, 0.53)	<0.0001
Systemic	0.34	(0.18, 0.63)	0.0007	0.20	(0.1, 0.4)	<0.0001
**Screening**						
No	2.4	(1.6, 3.6)	<0.0001	1.9	(1.2, 3.1)	0.009
Yes	Reference	Reference		Reference	Reference	

**Table 3:** Association between screening and the risk of 3-year all-cause mortality following hepatocellular carcinoma diagnosis among patients with viral hepatitis: Cox proportional hazard regression models, with survival times uncorrected and corrected for lead-time bias.

Characteristic	Univariate		
	HR	95% CI	*p*
No adjustment for lead-time bias			
**Screening**			
No	2.4	(1.6,3.6)	<0.0001
Yes	Reference	Reference	
Mean Sojourn Time = 70 days			
**Screening**			
No	2.19	(1.4, 3.3)	0.0002
Yes	Reference	Reference	
Mean Sojourn Time = 140 days			
**Screening**			
No	2.09	(1.4, 3.2)	0.0005
Yes	Reference	Reference	

## Data Availability

The datasets used and/or analyzed during the current study are available from the corresponding author upon reasonable request, subject to the satisfaction of institutional regulatory requirements.
